# Why medical students choose psychiatry - a 20 country cross-sectional survey

**DOI:** 10.1186/1472-6920-14-12

**Published:** 2014-01-15

**Authors:** Kitty Farooq, Gregory J Lydall, Amit Malik, David M Ndetei, Dinesh Bhugra

**Affiliations:** 1 Oxleas NHS Foundation Trust, 68 The Heights, Charlton, London SE7 8JH, UK; 2 Castel Hospital, Guernsey GY5 7NJ, UK; 3 Surrey and Borders NHS Trust, 18 Mole Business Park, Leatherhead, Surrey KT22 7AD, UK; 4 Africa Mental Health Foundation, 1st Floor Gakuo Court, Lower Hill Road, Off Haile Sellasie Avenue, P.O Box 48423, 00100 Nairobi, Kenya; 5 Mental Health and Cultural Diversity, PO25, Health Service and Population Research Department, Institute of Psychiatry, King’s College London, David Goldberg Centre, De Crespigny Park, London SE5 8AF, UK

**Keywords:** Psychiatry, Career choice, Medical student, Attitude to psychiatry, Stigma, Enrichment activity, Recruitment, Gender, Medical school selection

## Abstract

**Background:**

Recruitment to psychiatry is insufficient to meet projected mental health service needs world-wide. We report on the career plans of final year medical students from 20 countries, investigating factors identified from the literature which influence psychiatric career choice.

**Methods:**

Cross sectional electronic or paper survey. Subjects were final year medical students at 46 medical schools in participating countries. We assessed students’ career intentions, motivations, medical school teaching and exposure to psychiatry. We assessed students’ attitudes and personality factors. The main outcome measure was likelihood of specializing in psychiatry. Multilevel logistic regression was used to examine the joint effect of factors upon the main outcome.

**Results:**

2198 of 9135 (24%) of students responded (range 4 to 91%) across the countries. Internationally 4.5% of students definitely considered psychiatry as a career (range 1 to 12%). 19% of students (range 0 to 33%) were “quite likely”, and 25% were “definitely not” considering psychiatry. Female gender, experience of mental/physical illness, media portrayal of doctors, and positive attitudes to psychiatry, but not personality factors, were associated with choosing psychiatry. Quality of psychiatric placement (correlation coefficient = 0.22, p < 0.001) and number of placements (correlation coefficient =0.21, p < 0.001) were associated with higher ATP scores. During medical school, experience of psychiatric enrichment activities (special studies modules and university psychiatry clubs), experience of acutely unwell patients and perceived clinical responsibility were all associated with choice of psychiatry.

Multilevel logistic regression revealed six factors associated with students choosing psychiatry: importance of own vocation, odds ratio (OR) 3.01, 95% CI 1.61 to 5.91, p < 0.001); interest in psychiatry before medical school, OR 10.8 (5.38 to 21.8, p < 0.001); undertaking a psychiatry special study module, OR 1.45 (1.05 to 2.01, p = 0.03) or elective OR 4.28 (2.87- 6.38, p < 0.001); membership of a university psychiatry club, OR 3.25 (2.87 to 6.38, p < 0.001); and exposure to didactic teaching, OR 0.54 (0.40 to 0.72, p < 0.001).

**Conclusions:**

We report factors relevant to medical student selection and psychiatry teaching which affect career choice. Addressing these factors may improve recruitment to psychiatry internationally.

## Background

Psychiatry is facing a shortage of specialists, sometimes termed a “recruitment crisis” [1-4]. There is also a marked “Mental Health Gap” between the burden of mental health, substance misuse and neurological disorders worldwide, and resources [5]. The World Psychiatric Association (WPA) included recruitment to psychiatry as a major part of its 2008–2011 Action Plan [6]. There is a wide variation in psychiatric training opportunities worldwide [7-9] which is reflected in numbers of places available. For example with over 24,000 medical graduates each year in the UK, there are fewer than 450 new places for training in psychiatry, thus even if adequate numbers of people are interested, they may not be able to find jobs [10].

Reasons for low recruitment levels are varied. Studies of psychiatry career choice have generally been conducted at national levels [11-24], but not yet at a multinational level. The vast majority of literature identified in our systematic review was from the United States (36%) and UK (25%) [25]. These studies have examined personal and experiential factors as reasons why medical students may or may not choose psychiatry as their first career option.

Potential psychiatrists may decide on a career in psychiatry before, during, or after medical school. Factors associated with choosing psychiatric careers include exposure to psychiatrists or mental illness prior to medical school, and subsequent exposure to positive clinical experiences and “enrichment activities” such as electives and research to confirm their interest during medical school [26-28]. The summary of factors already identified as influencing career choice is shown in Table 1 and the effect of enrichment activities in our sample is shown in Table 2. Our literature search to identify these factors took included papers from 1999 onwards to try to reduce the impact of variation over time.

**Table 1:** Published associations with choice of psychiatry as a career

**Factor**	**Effect**
Gender	More women students than men choose psychiatry
Influences on choosing medicine	Personal or family experience of mental illness is more common
Pre-medical school qualifications	Students are no more likely to have previous degree, but, if they do, are more likely to have an arts or humanities background
School leaving qualifications	Students are more likely to have studied arts or humanities at school
Pre-medical school career choice	Interest in psychiatry prior to medical school is maintained in the final year
Quality of teaching	Positive correlation between teaching placement quality and ATP score
Teaching exposure	Positive correlation between the number of teaching exposures and ATP-18, and likelihood of choosing psychiatry
Enrichment activities	Exposure to enrichment activities (special study modules, electives and university psychiatry clubs) increases the likelihood of students choosing psychiatry
Stage of exposure	Early or late clinical exposure during medical school makes no difference
Duration of exposure	Positive correlation between placement length and likelihood of choosing psychiatry
Setting of exposure	Experience of outpatient or specialist services increases choice of psychiatry than inpatient services
Types of patients seen	Experience of people in recovery or people seeking help are more likely to choose psychiatry
Responsibility during placement	Positive correlation between higher levels of responsibility for patient care and likelihood of choosing psychiatry
Perception of psychiatry compared to other fields	Psychiatry is seen as having better job prospects, more flexibility, higher burnout and lower prestige
ATP-18*	ATP-18 scores are positively correlated with choosing psychiatry
Personality traits	Those choosing psychiatry will score higher on measures of neuroticism and openness compared to those choosing other fields

*ATP: Attitude to Psychiatry Scale.

**Table 2:** Enrichment activities and relation to likelihood of choosing psychiatry

**Factor**	**Category**	**Unlikely N (%)**	**Likely N (%)**	**P-value**
Psychiatry special study module	No	1387 (82%)	311 (18%)	0.03
	Yes	297 (77%)	89 (23%)	
Research experience in psychiatry	No	1632 (82%)	368 (18%)	<0.001
	Yes	52 (62%)	32 (38%)	
University psychiatry club	No	1630 (82%)	351 (18%)	<0.001
	Yes	54 (52%)	49 (48%)	
National psychiatry club	No	1667 (81%)	391 (19%)	0.05
	Yes	17 (65%)	9 (35%)	
Psychiatry elective	No	1561 (84%)	304 (16%)	<0.001
	Yes	123 (56%)	96 (44%)	
Problem-based learning scenarios	No	1253 (81%)	298 (19%)	0.97
	Yes	431 (81%)	102 (19%)	
e-learning	No	1556 (81%)	368 (19%)	0.79
	Yes	128 (80%)	32 (20%)	
Psychiatry simulation teaching	No	1494 (82%)	342 (19%)	0.07
	Yes	190 (77%)	58 (23%)	
Visits to secure units/prisons	No	1204 (81%)	286 (19%)	0.99
	Yes	480 (81%)	114 (19%)	
Lectures/tutorials	No	584 (78%)	166 (22%)	0.01
	Yes	1100 (82%)	234 (18%)	
Specialist placement	No	1203 (81%)	279 (19%)	0.50
	Yes	481 (80%)	121 (20%)	
Optional extras on course	No	1590 (82%)	357 (18%)	<0.001
	Yes	94 (69%)	43 (31%)	

Generally around 3% of students overall select psychiatry as a career [29], and those with positive attitudes towards mental health were around three times more likely to do so [18]. It is useful to understand what factors play a role especially across nations as doctors are global citizens and likely to move around more frequently.

In this study we focused on medical students because this group reflects medical school selection processes and the effects of psychiatry teaching and clinical exposure. This group may also be influenced by factors which medical educators and policy makers can address with sufficient evidence and will.

### Objectives

To report on career plans of final year medical students in 46 medical schools from 20 countries, with reference to factors identified from the literature as influencing psychiatry as a career choice. The null hypothesis was that these established factors predicting choice of psychiatry will have no association with respondents’ career choices.

## Methods

### Design and sample

This study used a quantitative cross-sectional design. Using an existing network of early career psychiatrists, high, low and middle-income countries were identified to obtain a sample of countries with a wide variation in mental health care systems and health care spending. An opportunistic sample of medical schools was selected in each country. The whole population of final year students within each school was invited to participate in the study.

### Measurements

We developed an online questionnaire which included questions on demographics, influences in choosing medicine, pre-medical school qualifications, pre-medical school career choice, and quality of teaching, clinical exposure, enrichment activities, and reported clinical placement responsibility during medical school. We included the ATP-18 (Attitude to Psychiatry Scale [29], and International English Big 5 Mini-Markers scale [30] which have been validated in a number of populations.

The questionnaire was piloted in the UK in November 2009, using a sample drawn from student members of the Royal College of Psychiatrists, and is available on request. The study was performed in different countries between March 2010 and June 2011 taking into account large variations in the timescales of ethics applications, and different timings between Northern & Southern Hemisphere term-times.

Where the local language was considered by local investigators to be preferable to English, the instruments were translated, back-translated, checked for accuracy and agreed translations were used. The final questionnaire was either sent out directly by a secure online survey service, or through email via a participating member of the medical school faculty. The exceptions were Japan and the African Mental Health Forum countries (excluding South Africa), which chose to use paper versions of the survey due to lack of Japanese language support from online tools, and limited internet access respectively.

Reminders were sent out at 4 and 8 weeks, and a standard protocol for maximising the response rate was followed including a short presentation to students before the reminders were sent out, and the use of local publicity and student representatives to raise the study profile and to prompt responses.

Ethical approval, where required, was obtained in each country’s relevant institution. In the UK this was granted by each participating medical school’s ethics panel.

### Power calculation

Previous research [29] had suggested that around 3% of students select psychiatry as a career; and that those medical students with positive attitudes towards psychiatry were around three times more likely to choose psychiatry as a career. To achieve a threefold difference it was assumed that 1.5% of those with negative attitudes, and 4.5% of those with positive attitudes would choose psychiatry as a career. To detect this level of difference with a 5% significance level and 90% power would require 1486 responses. It was originally envisaged that 3000 questionnaires would be distributed globally assuming a 50% response rate. However the response rate was low (17%), so a greater number (9373) was distributed to increase the power.

### Statistical methods

Categorical variables were summarised by the number and percentage of subjects in each category. Continuous variables were summarised using the mean and standard deviation.

Statistical analyses were performed using the program Stata v10. Significance level was set at 5% (p < 0.05, 2-sided).

The primary analysis examined factors associated with the students reporting a choice of career in psychiatry. This variable was originally measured on a 5-point Likert scale (no way, unlikely, possible, seriously consider and definitely) and was taken from the instrument designed by Feifel [31]. For the purposes of analysis, this scale was reduced to a binary outcome, with students either being “unlikely” to specialise in psychiatry (no way, unlikely, possible), or “likely” to specialise in psychiatry (seriously consider, definitely). It was not considered appropriate to treat this outcome as a continuous variable for two main reasons. Firstly this would assume equal spacing of the categories, which is not necessarily the case with a subjective ordinal variable. Secondly if it was assumed that this variable is continuous, then the analysis methods would assume a normal distribution for this measure. This is not the case here as the values are skewed towards the lower end of the scale (i.e. not choosing psychiatry).

The association between categorical variables and this measure was assessed using the Chi-square test. Differences in continuous variables between the two groups were examined using the unpaired t-test.

An additional analysis examined the association between ATP-18 score and likelihood of choosing psychiatry, and perception of psychiatry relative to other fields. All variables were measured on either ordinal or continuous scales, using Spearman’s rank correlation.

Personality traits were examined using the International English Mini-Markers scale, with permission from the author [30]. Each item was scored as being either ‘inaccurate’, ‘neutral’ or ‘accurate’. A score of 1–3 was attributed to each of these three categories. The scoring of personality traits that were negatively phrased was done using reverse coding. The total score for each trait was calculated, giving a value between 8 and 24. A higher value implied that the students associated themselves with that trait.

Differences in personality trait scores between students seriously considering specialising in psychiatry and those not seriously considering were assessed using the unpaired t-test.

We considered carefully whether to correct for multiple testing. We concluded that it was not necessary to employ this method in this situation where we were examining the effects of separate variables.

### Multilevel logistic regression

It was predicted that responses from students in the same countries would be more similar than responses from students in different countries. To allow for this data structure, and the binary nature of the outcome variable (choice of psychiatry or not), the analysis was performed using multilevel logistic regression methods.

The original analysis examined the effect of a large number of variables upon the main outcome. To avoid overloading the regression model, this analysis was restricted to variables shown to have a probable level of association with the outcome from the initial analyses (variables with a p-value of <0.2, Tables 2 and 3), and a backwards selection procedure was employed. The latter involved removing the non-significant terms one at a time until only the statistically significant variables remained. Initially the ATP-18 score was omitted from the analysis, because data on this score was only available for around half of participants. The inclusion of this variable would have restricted the number of students included in the analysis. However, subsequently the effect of this variable was also examined in the regression because attitudes to psychiatry are linked to career choice [18].

**Table 3:** Medical school clinical exposure factors and relation to likelihood of choosing psychiatry

**Factor**	**Category**	**Unlikely N (%)**	**Likely N (%)**	**P-value**
Time of psychiatry placement	First half	93 (85%)	17 (15%)	0.45
	3rd quarter	543 (82%)	123 (18%)	
	Final quarter	715 (80%)	179 (20%)	
Weeks of psychiatry teaching	≤ 5 weeks	648 (82%)	145 (18%)	0.43
	6-10 weeks	298 (79%)	81 (21%)	
	11+ weeks	278 (80%)	70 (20%)	
Main psychiatry placement setting	Inpatient	984 (82%)	216 (18%)	0.13
	Outpatient	202 (77%)	61 (23%)	
	Specialist	155 (79%)	40 (21%)	
Seen people in crisis	No	652 (82%)	146 (18%)	0.06
	Yes	620 (78%)	175 (22%)	
Seen people in recovery	No	376 (78%)	105 (22%)	0.27
	Yes	896 (81%)	216 (19%)	
Seen prodromal symptoms	No	913 (80%)	224 (20%)	0.48
	Yes	359 (79%)	97 (21%)	
Seen patients motivated to seek help	No	532 (80%)	130 (20%)	0.67
	Yes	740 (79%)	191 (21%)	
Seen acutely unwell patients	No	522 (82%)	111 (18%)	0.04
	Yes	750 (78%)	210 (22%)	
Seen people with chronic symptoms	No	159 (880%)	39 (20%)	0.87
	Yes	1113 (80%)	282 (20%)	
Level of responsibility during placement	None	507 (79%)	135 (21%)	<0.001
	Asked opinion	417 (83%)	83 (17%)	
	First clerking	215 (83%)	45 (17%)	
	Assess risk/do therapy	135 (69%)	60 (31%)	

## Results

**Figure 1:**
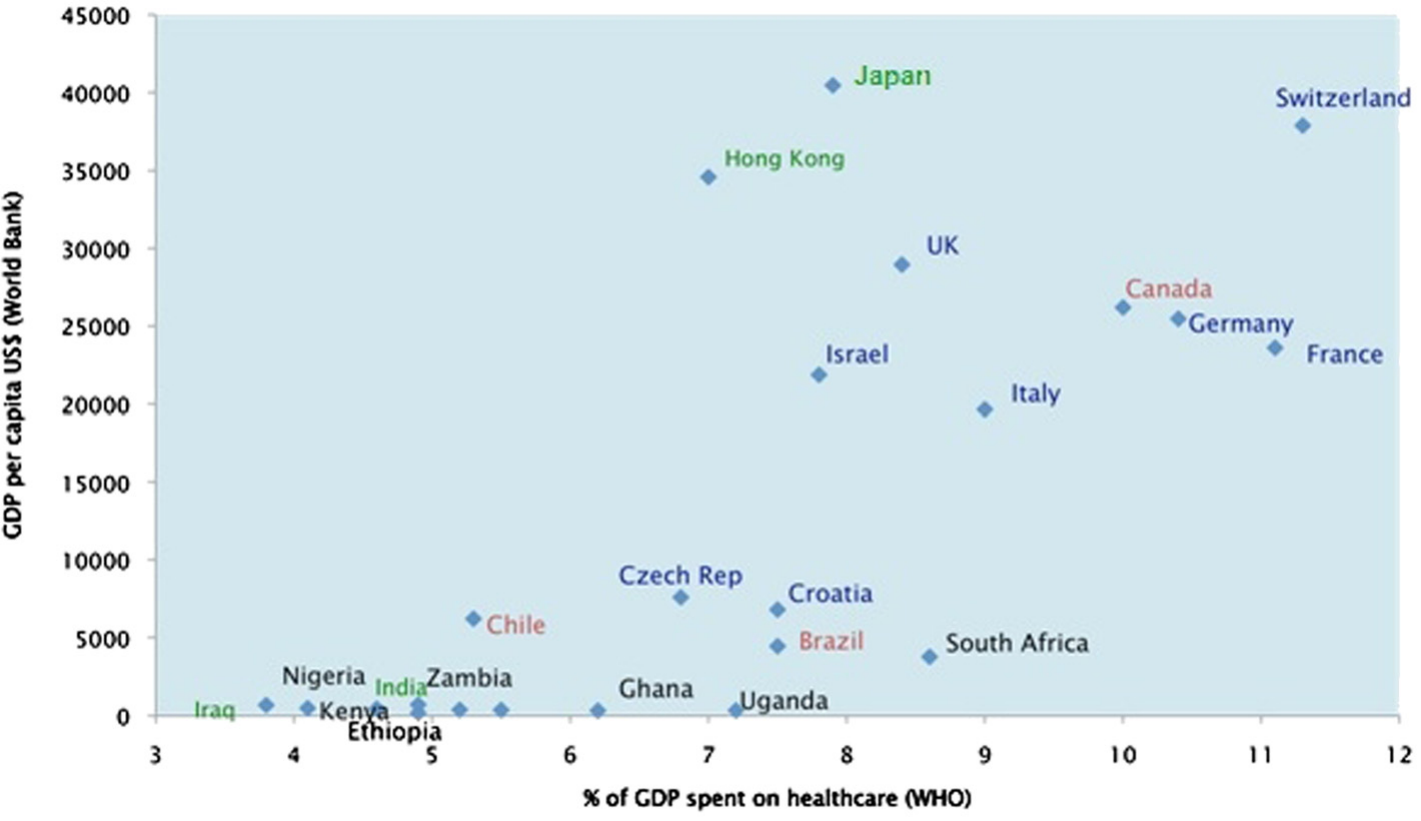
Participating countries’ 2011 Gross Domestic Product (GDP) per capita vs. percentage healthcare spend.

A total of 2198 students from 46 medical schools in 20 countries consented to take part in the survey. The number of responses per country ranged from 9 (Tanzania) to over 300 (Germany). The spread of countries is represented in Figure 1, using 2011 World Bank (http://data.worldbank.org/indicator/) and World Health Organization (WHO) Global Health Expenditure (http://apps.who.int/nha/database/DataExplorerRegime.aspx) data to illustrate gross domestic product (GDP) per capita and percentage of GDP spent on healthcare. A summary of the number of responses from each country, and demographic details are shown in Table 4.

**Table 4:** Country response rates, demographics, and likelihood of choosing psychiatry

**Country**	**Number of responses**	**Sample size**	**Response rate %**	**Age, mean (SD)**	**International student N (%)**	**Ethnic minority N (%)**	**Male gender N (%)**	**No way would choose psychiatry N (%)**	**Seriously considering psychiatry N (%)**	**Definitely considering psychiatry N (%)**	**Likely to choose psychiatry N (%)**‡
Croatia	136	385	35%	24.6 (1.7)	O (0%)	5 (4%)	37 (28%)	37 (28%)	21 (16%)	8 (6%)	29 (22%)
Czech Republic	78	239	33%	25.2 (1.0)	14 (21%)	6 (9%)	22 (32%)	22 (31%)	6 (8%)	5 (7%)	11 (15%)
France	114	446	26%	24.8 (1.1)	0 (0%)	(*)	35 (32%)	45 (40%)	15 (13%)	13 (12%)	28 (25%)
Italy	104	360	29%	25.2 (1.2)	0 (0%)	(*)	38 (37%)	41 (39%)	15 (14%)	6 (6%)	21 (20%)
Israel	173	534	32%	27.9 (2.9)	16 (9%)	11 (6%)	83 (48%)	40 (23%)	35 (20%)	5 (3%)	40 (23%)
Germany	323	2000	16%	23.8 (3.9)	6 (2%)	18 (6%)	98 (31%)	87 (27%)	69 (22%)	8 (3%)	77 (24%)
Switzerland	60	221	27%	25.8 (2.2)	4 (7%)	9 (16%)	22 (39%)	18 (31%)	8 (14%)	2 (3%)	10 (17%)
UK	291	1810	16%	24.7 (4.6)	22 (8%)	74 (27%)	99 (37%)	65 (23%)	53 (19%)	6 (2%)	59 (21%)
Canada	127	592	21%	24.6 (4.1)	2 (2%)	30 (25%)	26 (21%)	21 (17%)	21 (17%)	5 (4%)	26 (21%)
Brazil	13	307	4%	24.8 (2.6)	0 (0%)	2 (17%)	8 (67%)	9 (75%)	0 (0%)	1 (8%)	1 (8%)
Chile	40	440	9%	24.4 (1.9)	0 (0%)	0 (0%)	22 (58%)	14 (36%)	9 (23%)	3 (8%)	12 (31%)
Hong Kong	45	250	18%	23.7 (2.1)	1 (2%)	0 (0%)	24 (53%)	5 (11%)	8 (18%)	4 (9%)	12 (27%)
India	66	263	25%	22.2 (1.1)	8 (13%)	11 (17%)	24 (38%)	12 (19%)	5 (8%)	1 (2%)	6 (9%)
Iraq	71	296	24%	22.7 (1.0)	23 (35%)	20 (31%)	31 (48%)	13 (19%)	14 (21%)	8 (12%)	22 (33%)
Japan	145	200	73%	22.9 (2.2)	(*)	(*)	112 (79%)	37 (29%)	10 (8%)	1 (1%)	11 (9%)
Kenya	182	254	72%	24.8 (1.8)	28 (18%)	1 (1%)	75 (45%)	5 (3%)	7 (4%)	12 (7%)	19 (12%)
Nigeria	95	104	91%	24.3 (2.1)	8 (9%)	6 (8%)	48 (55%)	18 (24%)	3 (4%)	3 (4%)	6 (8%)
South Africa	70	360	19%	23.9 (1.6)	6 (9%)	38 (55%)	27 (40%)	19 (27%)	8 (11%)	1 (1%)	9 (13%)
Tanzania	9†	(*)	(*)	26.7 (1.7)	1 (13%)	1 (11%)	4 (44%)	2 (33%)	0 (0%)	0 (%)	0 (0%)
Uganda	56	74	76%	24.3 (1.5)	2 (4%)	5 (9%)	34 (61%)	17 (34%)	0 (0%)	1 (2%)	1 (2%)
Combined	2189	9135	24%	24.6 (3.1)	141 (7%)	237 (14%)	869 (41%)	527 (25%)	307 (15%)	93 (4%)	400 (19%)

Key: (*) Information not available from this country (**†**) Not included in response rate data (**‡**) Combining ‘definitely’ and ‘seriously considering’ scores.

Respondents had a mean age of 24 years. The majority (93%) were home rather than international students, although this overall figure hid a wide variation between individual countries. 14% of students were from an ethnic minority within their country of study (range 0% to 55%). 41% of the students were men, although this also varied by country (range 21% to 79%). South American, Asian and African countries had a higher percentage of men respondents, whist the European countries and Canada had a higher proportion of women responding to the survey.

### Likelihood of specialising in psychiatry

**Figure 2:**
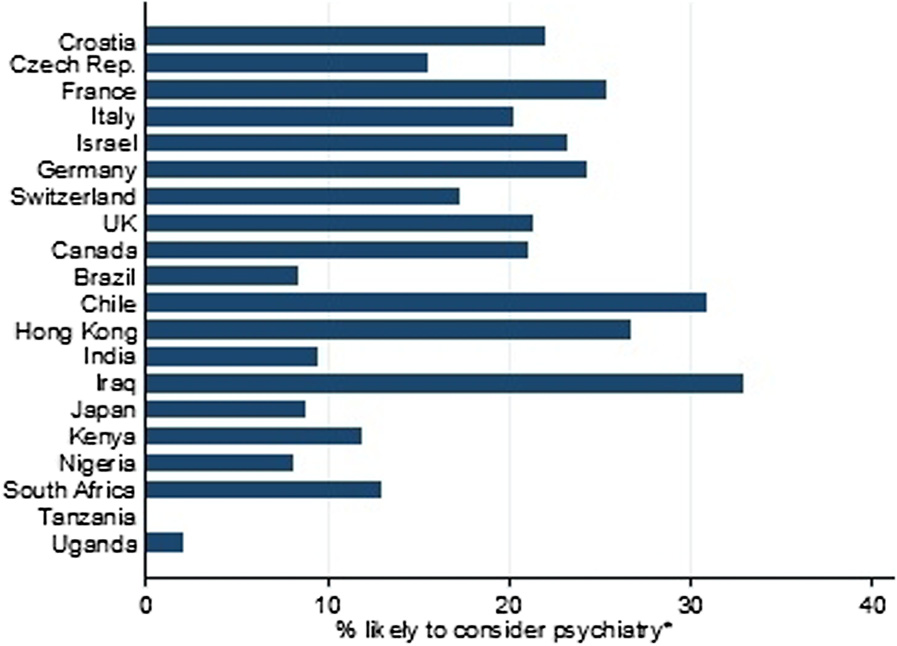
**Variation in percentage of medical students seriously or definitely considering a career in psychiatry by country (alphabetical order).** Key: *Combining ‘definitely’ and ‘seriously considering’ scores.

The primary analysis examined the likelihood of participants specialising in psychiatry. A summary of the proportion of respondents and their reported likelihoods of specialising in psychiatry by country along with demographic findings is shown in Table 4. The overall results suggested that 4.5% of respondents were “definitely” considering specialising in psychiatry, and another 15% were “seriously considering” specialising in the field, giving a combined “likely” total of 19%. The variation in percentage of medical students seriously considering or definitely considering a career in psychiatry (combining ‘definitely’ and ‘seriously considering’ scores) is shown in Figure 2.

Interestingly there was a higher likelihood of choosing psychiatry in the European countries, with a lower likelihood in African countries. No students in Tanzania (although based on small numbers) and only 1% in Uganda were likely to choose psychiatry. By comparison, up to 31% of students sampled in Chile and 33% in Iraq were likely future psychiatrists.

### Pre-medical school factors associated with choosing psychiatry

The first set of analyses examined the association between demographic details of the participants and choosing psychiatry. Women were more likely to choose psychiatry than men (21% vs. 16%, p = 0.007). There were no differences by ethnicity, social class or urbanicity. Not surprisingly personal or family experience of physical illness (p = 0.001) and mental illness (p < 0.001) were likely to influence choice of speciality. Four factors that influenced the choice to study medicine were also found to be significantly associated with specialising in psychiatry: students’ own vocation (p = 0.02), the media portrayal of doctors/medicine (p = 0.03), and past experience of physical (p = 0.001) and mental illness (p < 0.001).

No association was found between the students’ qualifications before medical school and subjects studied at school, and their likelihood of specialising in psychiatry.

Preference of speciality when entering medical school was strongly associated with subsequent speciality choice. Perhaps unexpectedly, 78% of those whose initial choice was psychiatry when entering medical school remained likely to consider specialisation in the subject by the end.

### Medical school factors associated with Choosing Psychiatry

#### Teaching methods, student ratings, and enrichment activities

Table 2 illustrates the impact of teaching methods and activities on choice of a career in psychiatry.

Almost half (48%) of those involved in a psychiatry club at university were likely to consider a career in the field, compared to only 18% who did not attend such a club. Students who had exposure to psychiatry lectures and tutorials were slightly less likely to consider a specialisation in psychiatry, whilst those with optional extras on the course were more likely to consider psychiatry as a career.

Interestingly, those who were taught psychology were less likely to consider specialising in psychiatry compared with other subjects.

### Clinical exposure factors

The majority of clinical exposure factors were not associated with likelihood of choosing psychiatry (Table 3). However, those students exposed to acutely unwell patients were more likely to consider psychiatry as was the degree of clinical responsibility given to them especially risk assessment, or therapy. Nearly one third (31%) of the group with most responsibility were likely to consider psychiatry compared to one fifth (around 20%) for the remaining groups.

#### Attitudes towards psychiatry

Not surprisingly students with a positive attitude score were more likely to consider psychiatry, with 23% of this group considering it as a speciality, compared to only 12% of those with a negative attitude. Results of attitudes to psychiatry are shown in Table 5.

**Table 5:** Rating of psychiatry relative to other fields, ATP-18 scores and relation to career choice

**Factor**	**Category**	**Unlikely to choose psychiatry n (%)**	**Likely to choose psychiatry n (%)**	**P-value**
Psychiatry relative other specialities ^(*)^	Worse	151 (83%)	32 (17%)	0.30
	Similar	414 (77%)	123 (23%)	
	Better	232 (79%)	66 (22%)	
ATP score ^(^†^)^	-	60.7 (6.1)	64.4 (7.4)	<0.001
ATP score	Negative attitude	415 (88%)	56 (12%)	<0.001
	Positive attitude ^(^‡^)^	429 (77%)	125 (23%)	

(*) Combined variable based on 8 individual criteria, (**†**) Mean (standard deviation) reported, (**‡**) Positive attitude defined as score >60.

#### Teaching exposure, attitudes & career choice

The association between ATP score and two other factors (quality of psychiatric placement, and number of exposures to psychiatric teaching) was examined using Spearman’s rank correlation (Table 6). Quality of psychiatric placement was significantly associated with career choice and number of psychiatric teaching placements.

**Table 6:** Quality & quantity of teaching & ATP-18 scores

**Factor**	**Correlation coefficient**	**P-value**
Quality of psychiatric placement	0.22	<0.001
Number of exposures to psychiatric teaching/placements	0.21	<0.001

#### Personality traits

Interestingly there was no association between self-rated personality factors and likelihood of choosing psychiatry on the International English Mini-Markers scale (Table 7).

**Table 7:** Personality traits and likelihood of choosing psychiatry

**Personality trait (International English Mini-Markers scale)**	**Unlikely mean (SD)**	**Likely mean (SD)**	**P-value**
Extraversion	17.4 (3.7)	17.3 (3.7)	0.84
Openness	18.3 (3.3)	18.6 (3.7)	0.32
Neuroticism	16.0 (3.7)	15.7 (4.0)	0.55
Conscientiousness	17.5 (4.2)	17.4 (4.3)	0.72
Agreeableness	21.0 (3.3)	21.1 (3.5)	0.76

#### Logistic regression

Multilevel logistic regression was used to examine the joint effect of various factors upon likelihood of choosing a career in psychiatry. A backwards selection procedure was used to retain only the statistically significant variables. This final regression model is based on 1732 respondents. This gives the odds of being likely to choose a career in psychiatry in each category relative to the odds in a baseline category.

Six factors were found to be associated with students being likely to pursue a career in psychiatry (Table 8).

**Table 8:** Logistical regression factors in choosing a psychiatric career

**Variable**	**Category**	**Odds ratio (95% CI)**	**P-value**
Own vocation in choosing medicine	Negative influence	1	<0.001
	No/slight influence	1.49 (0.85, 2.61)	
	Important influence	3.01 (1.61, 5.91)	
Speciality choice on entering medical school	No speciality	1	<0.001
	Psychiatry	10.8 (5.38, 21.8)	
	Other speciality	1.13 (0.81, 1.59)	
Psychiatry special study module	No	1	0.03
	Yes	1.45 (1.05, 2.01)	
University psychiatry club	No	1	<0.001
	Yes	3.25 (1.94, 5.42)	
Psychiatry elective	No	1	<0.001
	Yes	4.28 (2.87, 6.38)	
Psychiatry lectures/ tutorials	No	1	<0.001
	Yes	0.54 (0.40, 0.72)	

Those who gave more weight to their own vocation were three times more likely to choose psychiatry. Those who had a clear idea that they wanted to study psychiatry when entering medical school were 11 times more likely to consider specialising in psychiatry compared to those who had no speciality choice on entering medical school.

Experience of a psychiatry special module made choice of psychiatry slightly more likely (45%) than those who had no exposure. A university psychiatry club (with guest lectures and social events) and psychiatry elective (dedicated to either clinical work or study elsewhere) were strongly associated with career choice of psychiatry (3 times and 4 times more likely respectively).

The odds of choosing psychiatry dropped to almost half as large for those who had experienced didactic teaching. The results suggest that the odds of choosing psychiatry were 46% higher for participants with a positive attitude compared to those with a negative attitude towards psychiatry.

## Discussion

This study set out to report on the career plans of final year medical students from 20 countries, with reference to factors identified from the literature as affecting psychiatry as a career choice.

### Strengths and limitations

The main strengths of the study are the large multi-country sample size, with sufficient power to detect differences of the key variables. It is the first international study of its kind, and the first study to look at relative influences of multiple well described factors on psychiatric recruitment.

The main limitation is the relatively low response rates from some countries. This might be explained by a number of factors in those countries including selection bias which may reflect different attitudes towards psychiatry. Conversely, the higher than expected endorsement of a psychiatric career choice in some countries may represent respondent selection bias. Different methods of administration of the questionnaire were unavoidable due to restricted Internet access in some countries, and lack of support for Japanese script on the online tools available. It is possible that this might have introduced bias, as reminders could be targeted at non-responders more easily using the online tools.

In the interests of developing a questionnaire sufficiently brief to improve response rates but remain useful, we decided *a priori* against exploring a number of areas of interest. These included cultural, linguistic, religious and other social variables (for a review see Rao et al. [32]. The absence of qualitative data also limits the ability to address larger-scale issues such cultural and professional stigma towards mental health and psychiatry.

ATP-18 data was missing in three countries due to an undetected online survey system error. The cross sectional design was chosen as the most practicable to cover a wide range of countries, therefore direction of effect and causality cannot be demonstrated. The wide variation between countries may be obscured by pooling the data, although we attempted to address this in the statistical analysis. The lack of data from countries like the USA and regions like Australasia are a further limitation to generalisability especially to these areas. These countries were not included in the initial study because of logistical reasons. The intention was to remedy this in a second phase of the study pending further funding. Lastly, we did not correct for multiple testing because we were examining the effects of separate variables. Uncorrected, this approach may lead to Type 1 errors.

### Interest in psychiatry

Our finding that 4.5% of the sample was definitely interested in a career in psychiatry is consistent with previous estimates [33-36], and suggests that the sample was representative. Our survey was timed in the second half of the students’ final year of medical school to capture their choices close to qualification. Previous research on decision stability [37-42] suggests that career decisions are becoming increasingly stable by this point. Between year 4 and qualification 56% will change their minds [38]. Of Goldacre and colleagues’ cohort of psychiatrists, 64% who had made their decision in the first year post qualification remained within the field after 10 years [43].

Estimates of the percentage of students who consider psychiatry as a career option but have not made a definite decision vary by country and methodology, for example 9% in the UK [44], 7.6% in Pakistan [45] and 33% in Israel [46]. Our combined finding was 15%, with geographical variation ranging from 0-33%, and thus broadly in line with previous results. The overall trend was for European countries and Canada to have higher rates for choosing psychiatry than African countries. Interestingly, Iraq has the highest rate at 33% which confirms previous reports that with the recognition of the psychiatric morbidity of war, post-conflict recruitment to psychiatry improves [47]. Funding and provision of mental health care in African countries with few psychiatrists, limited provision especially in rural areas and limited undergraduate teaching may affect recruitment although some attempts are being made to overcome these [48].

As mentioned above, funding, quality and availability of psychiatry training posts varies considerably worldwide, with only 68% of the world’s countries offering psychiatry training [7,8]. Even if adequate numbers of people are interested in psychiatry, their decisions may be influenced by training job availability and prospects. For example, less than 1.8% of new training posts in the UK are for psychiatry and competition ratios are nearly 5 applicant for every 1 post [10]. While outside the scope of this paper, it would be reasonable for countries to ensure optimal training (and specialist) job availability to capture those newly graduating doctors interested in psychiatry.

### Pre-medical school factors

The popularity of psychiatry in our study was greater for women, in line with trends seen in previous studies [49-51]. Flexible working patterns and role models may play a role in this decision. Our data did not confirm previous findings s that psychiatrists were more likely to come from urban areas [11].

There was a significant difference for strong internal vocation, and personal or family experience of physical or mental illness confirming previous findings [11,52,53]. There may also be an impact of societal stigma, as those choosing psychiatry placed less emphasis on the portrayal of doctors in the media on their choice of medicine as a career. Assessment of personal motivation for the career during interview and other selection procedures to medical school may select for students with these characteristics, so the importance of these factors for psychiatry recruitment should be recognised. [11,52,53].

Stigma, both cultural, personal and professional may negatively affect attitudes toward the mentally ill and psychiatry. This may in turn influence career choice before and during medical school; positive attitudes towards psychiatry increase the likelihood of choosing a career in the field [18]. However because undergraduate education appears to reduce stigma in several countries by improving attitudes teaching may indirectly improve psychiatry as a career prospect [54]. Furthermore, a recent meta-analysis showed that contact (with someone with mental illness, as may occur in clinical settings) was better than education at reducing stigma for adults [55].

More detail on the crucial area of stigma is beyond the scope of this paper. The interested reader is referred to a comprehensive review article on public and personal stigma [56] and professional stigma. We also recommend a recent meta-analysis on the public understanding of mental illness and stigma [57].

### Decision stability

2.7% our sample entered medical school with a desire to become a psychiatrist, and 78% of this group remained committed at year 5. This group of early-deciders made up 12% of the total of those who were interested in psychiatry by the final year of medical school. Thirty percent of those choosing psychiatry in year 5 had entered medical school with any speciality choice, and 70% did not have a preference. This is a lower figure than the 40% deciding before medical school found by Galeazzi [39] in a retrospective sample of psychiatry interns. The emphasis is different for the two groups: for early deciders it is ensuring a high conversion rate to psychiatry internship; and for those who develop their interest during medical school it is about standing out against other fields. Furthermore, reducing professional stigma (both from other doctors and mental health professionals themselves) during medical school may maintain this interest and reduce some of the attrition [18] This question warrants further exploration.

### Teaching exposure

Our data on attitudes from the ATP-18 was in line with previous studies [58-64], showing the majority of students had positive attitudes towards psychiatry, which were positively correlated to the quality and quantity of teaching (Table 6).

However, students’ ratings of the quality of their teaching were not associated with career choice, except for the unexpected finding that exposure to didactic teaching (lectures) appears to decrease recruitment to psychiatry. This may reflect that fact that this was the commonest method of delivery of teaching, with 1334 of the sample endorsing it. Students who were exposed to problem based learning or e-learning were no more likely to choose psychiatry, but there was a trend [p = 0.07] towards simulation teaching. It may also be that clinical exposure is rated more highly by students than didactic teaching as has been reported in several countries [65-67], or that clinical contact was better than education at reducing mental illness stigma [55]. It is also possible that the didactic training received by the students may be lacking in quality, depth, resources and other factors, perhaps painting an unflattering image of the field. Lastly, this counterintuitive finding may also represent Type 1 error. Because this result is not easily explained, and because teaching is a substantial part of the investment in the training of medical students, we recommend further studies on this subject.

### Enrichment activities

Specific activities such as research and special study modules may be used by students to test out potential career choices. Electives and university psychiatry societies were important for recruitment to psychiatry in our sample, and it is possible that these may be further improved to enhance recruitment [26-28].

### Clinical factors

Length and setting of clerkship made no difference to the likelihood of choosing psychiatry. Although previous work on clerkship setting has given conflicting results [68,69], and in an international context there will be inherent variation in settings, length and quality of clinical attachment did not make a difference per se but the degree of responsibility did.

It is possible that the current generation of students have a greater awareness of the potential for recovery from severe mental illness, so the stimulating exposure to acute work is more influential. It is possible that those already interested may seek out greater clinical exposure.

The association between the level of responsibility given to students during their clerkship and choice of career [70] is confirmed in the present study. There are local projects such as a student psychotherapy scheme [71], where medical students offer psychodynamic therapy under close supervision from staff which has increased recruitment to psychiatry [43].

## Conclusions

Overall, our findings are encouraging in that internationally there is a pool of 15% of students who would seriously consider psychiatry as a career. It is this group that is arguably the most critical to target at medical school in order to enhance recruitment. Our study has shown that there are specific aspects of undergraduate education that are associated with final year medical students choosing a career in psychiatry. Countries with lower rates of recruitment might look to their opposites to consider policy changes to improve local recruitment. Further studies are also needed in different countries to confirm prospectively specific national factors affecting recruitment to psychiatry.

## Competing interests

The authors declare no competing financial or non financial interests.

All authors have completed the Unified Competing Interest form at http://www.icmje.org/coi_disclosure.pdf (available on request from the corresponding author) and declare that (1) none have support from any company for the submitted work; (2) none have relationships with companies that might have an interest in the submitted work in the previous 3 years; (3) their spouses, partners, or children have no financial relationships that may be relevant to the submitted work; and (4) all have no non-financial interests that may be relevant to the submitted work.

## Authors’ contributions

The study was led by KF, GL, AM and DB. The article was drafted by GL and KF. All authors contributed to the submitted article. The guarantor is DB. Ethical approval, where required, was obtained in each country’s relevant institution. In the UK ethical approval was granted by the ethics panels at each participating medical school. An independent statistician, PB, was employed in the study design and data analysis. All authors read and approved the final manuscript.

## Pre-publication history

The pre-publication history for this paper can be accessed here:

http://www.biomedcentral.com/1472-6920/14/12/prepub
